# Reasons for First Dental Visit Among Infants and Toddlers in Bucharest, Romania—A Cross-Sectional Study

**DOI:** 10.3390/children13070964

**Published:** 2026-07-21

**Authors:** Aneta Munteanu, Sabina Teodorescu-Drăghicescu, Mihaela Tănase, Diana Daciana Zmărăndache, Amiel Stroianu, Ioana Andreea Stanciu

**Affiliations:** 1 Pediatric Dentistry Department, Faculty of Dentistry, Carol Davila University, 041292 Bucharest, Romania; aneta.munteanu@umfcd.ro (A.M.); sabina.teodorescu@rez.umfcd.ro (S.T.-D.); ioana.stanciu@umfcd.ro (I.A.S.); 2 Pediatric Dentistry Unit, Barzilai Medical University Centre, Ashkelon 7830604, Israel; stroianu@gmail.com

**Keywords:** first dental visit, infants, toddlers, caries experience

## Abstract

**Highlights:**

**What are the main findings?**

- Approximately 75% of children did not attend their first dental visit at the recommended age. 57.14% of them were diagnosed with caries, and those living in rural areas exhibited significantly higher decay rates than their urban peers.
- Only 6.68% of affected children had non-cavitated lesions, while 50.58% were already diagnosed with complicated caries. 55.31% of children received a routine check-up with oral hygiene and dietary counseling at their first dental visit.

**What are the implications of the main findings?**

- At their first dental visit, many children were found to have advanced dental disease requiring invasive treatment rather than preventive care.
- There is a need to increase parental awareness of the importance of early dental visits, particularly in rural areas and among children with special needs.

**Abstract:**

Background: In Romania, data regarding the oral health of infants and toddlers is scarce. This study aimed to analyze the reasons for the first dental visit among children aged 3 or under and to assess their oral health status and the therapeutic methods applied. Methods: A retrospective cross-sectional study was conducted on 602 children (339 boys and 263 girls), aged ≤ 3 years, who were referred for their first dental visit to the Pediatric Dentistry Department, Faculty of Dentistry, Carol Davila University, Bucharest, Romania. Results: The main reasons for referral were routine check-up (28.23%), asymptomatic carious lesions (21.92%), abscess or swelling (18.43%), and dental trauma (14.78%). Caries was diagnosed in 344 children; 23 (6.68%) had only non-cavitated lesions, 157 (45.64%) had uncomplicated cavitated lesions, and 174 (50.58%) already had complicated caries. Mean values of caries experience indices were significantly higher in rural children (dmf-t = 4.33 ± 4.13; dmf-s = 9.16 ± 11.34) than in urban children (dmf-t = 2.67 ± 3.61; dmf-s = 5.62 ± 8.86) (*p* = 0.000). The most frequent treatments were routine check-up with oral hygiene and dietary instructions (55.31%), pulp treatments (19.91%), and restorative procedures (15.1%). Conclusions: Many infants and toddlers were referred later than the recommended age and required therapeutic rather than preventive care. More than half had carious lesions, and many of those were already complicated. Significant disparities were observed between rural and urban children from this study group. Targeted public health initiatives are needed to improve early access to dental care.

## 1. Introduction

Multiple national health care organizations—including the American Academy of Pediatric Dentistry (AAPD), the American Dental Association and the American Association of Public Health Dentistry—recommend that a child’s first dental visit should occur within six months after the eruption of the first primary tooth and no later than 12 months of age [1,2,3]. The main purposes of an early dental visit are to educate parents about oral health, teething management, oral habits, injury prevention, and the early detection of caries, as well as to establish a positive relationship between the child and the pediatric dentist, in accordance with the AAPD’s “dental home” concept [1,4]. A dental home is defined as an ongoing relationship between the dentist and the patient in which all aspects of oral health care are delivered in a safe, individualized, accessible, and family-centered manner [1].

According to the AAPD, there is strong clinical evidence supporting the efficacy of early professional dental care [1]. Studies have shown that children who visit the dentist at an early age develop fewer caries than those who attend later. Furthermore, early lesions are more likely to be incipient, whereas dental disease in older children tends to be more severe. Long-term findings indicate that infants and toddlers who receive early preventive and therapeutic care experience an overall improved quality of life. Early childhood caries—one of the most prevalent yet preventable diseases—may lead to serious complications, affecting future oral health, self-esteem, and cognitive development [5,6,7,8,9,10].

However, several studies show that the age and reasons for a child’s first dental visit vary widely. Recent epidemiological studies conducted worldwide indicate that only a small percentage of children visit the dentist before one year of age [11]. Large surveys from the USA, Australia, and Poland report that most children have their first dental visit after the age of three [12,13,14,15].

In many cases, the first dental visit is prompted not by preventive care or the establishment of a dental home, but by pain or acute symptoms. Studies by Bulut & Bulut (2020) and Munteanu et al. (2025) found that pain was the most common reason for the first dental visit among children aged 0–5 years [4,16].

Assessing the age and reasons for the first dental visit is essential for raising public awareness and provides valuable data for clinicians and public health planning [4]. In Romania, only a few studies have examined the oral health of young children [16,17,18]. These studies, conducted among children aged 0–5 years, reported a high prevalence of caries, elevated caries experience indices, and a large proportion of untreated lesions. Mean caries experience values were higher among children from rural areas and among those with special needs [16,17,18].

Considering these aspects, the present study aimed to analyze the reasons for the first dental visit among infants and toddlers and to assess their oral health status and the therapeutic methods applied.

## 2. Materials and Methods

A retrospective observational cross-sectional study was conducted on a sample of 602 children (339 boys and 263 girls), aged between 1 month and 3 years 11 months, who were referred for their first dental visit to the Pediatric Dentistry Department, Faculty of Dentistry, Carol Davila University, Bucharest, Romania, between January 2020 and December 2025. For all included children, this was their first-ever dental visit.

The study was approved by the Ethics Committee of “Carol Davila” University of Medicine and Pharmacy of Bucharest (Protocol No. 8522/2.04.2026). Parents provided written informed consent prior to the oral examination.

**Inclusion criteria** were:

- children aged ≤ 3 years;
- children with or without special needs;
- children with complete dental records.

**Exclusion criteria** were:

- children older than 3 years;
- children with incomplete or uncertain dental records.

Data extracted from the dental files included demographics (age, sex, residence in urban or rural areas), the main reason for referral, general health status, dental health status (caries-free or presence of decayed teeth), and the treatment performed during the first visit.

Sample distribution according to age, gender, residence, general health status, reasons for referral, and treatment methods was analyzed. The prevalence of oral and dental conditions identified at the first dental visit was determined, and mean values of caries experience indices (dmf-t, dmf-s) were calculated.

Diagnostic criteria were defined as follows:

- **Non-cavitated caries:** no cavitation or loss of enamel surface continuity; lesion confined to enamel; no detectable softening or dentin exposure; tooth surface may appear white, opaque, or brownish but remains intact.
- **Cavitated caries:** clear loss of tooth structure; dentin clinically visible; cavity accessible for tactile examination; no signs of pulpal involvement (otherwise classified as complicated caries).
- **Complicated caries:** pulpal involvement; deep caries with spontaneous pain or visible pulp exposure; simple/complicated lesions with apical periodontitis; tooth may be non-vital or asymptomatic; swelling, purulent discharge, or fistula may be present.
- **Dental trauma:** enamel fracture (loss of enamel only); enamel–dentin fracture (loss of enamel and dentin without pulp exposure); complicated crown fracture (pulp exposure); root fracture; luxation injuries (concussion, subluxation, extrusion, lateral luxation, intrusion); avulsion (complete displacement of the tooth from the socket).
- **Treatment categories:** check-up, GIC restoration, interim filling, desensitizing pulp therapy, abscess drainage, antibacterial mouthwash, professional dental cleaning, referral for extraction or treatment under general anesthesia, and endodontic therapy. Each child was assigned to one main treatment category.

Examinations were performed by three clinicians (A.M., M.T., I.A.S.) from the Pediatric Dentistry Department, using a standardized clinical file. Inter-rater reliability was assessed using Cohen’s kappa coefficient after examining 30 patients, with all kappa values above 0.9.

Data were analyzed using SPSS 20.0 for Windows (SPSS Inc., Chicago, IL, USA). Descriptive statistics, including frequencies and percentages, were reported. The Chi-square test was used to evaluate group differences in categorical variables. Normality of dmf-t and dmf-s values was assessed using the Shapiro–Wilk test and Q–Q plots. Welch’s t-test was applied to compare mean caries experience indices between groups due to unequal sample sizes and variances. Statistical significance was set at *p* = 0.05.

## 3. Results

### 3.1. Study Group Distribution by Age, Sex, Residence, and General Health Status

**Figure 1:**
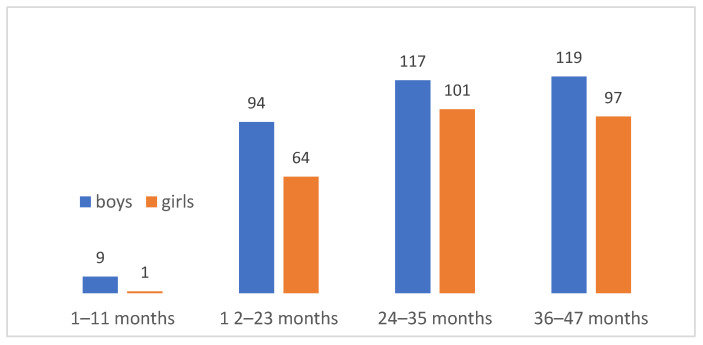
Age and sex distribution of the study sample (n = 602 children).

Children in the study were aged between 1 and 47 months (mean age = 2.52 ± 0.83 years). Most children—nearly 75%—were referred at ages 2 or 3 (Figure 1).

A total of 77.41% of the 602 children lived in urban areas, while 22.59% resided in rural areas. Most children (95.18%) were healthy, whereas 4.82% (n = 29) had general medical conditions or genetic syndromes.

### 3.2. Reasons for the First Dental Visit

The most common reason for the first dental visit was a routine dental check-up, followed by the presence of asymptomatic carious lesions (Table 1).

### 3.3. Caries Experience at the First Dental Visit

Caries (non-cavitated and/or cavitated) was diagnosed in 344 children (57.14%). Caries prevalence increased markedly with age: 10% in infants under the age of 1, 39.87% in 1-year-olds, 50.45% in 2-year-olds, 78.70% in 3-year-olds.

Caries prevalence was slightly higher in girls (61.22%) than in boys (54.87%), but the difference was not statistically significant (χ^2^ = 2.445, df = 1, *p* = 0.118).

Among the 344 children with caries: 23 (6.68%) had only non-cavitated lesions, 157 (45.64%) had uncomplicated cavitated lesions, and 174 (50.58%) had complicated caries.

Mean caries experience indices increased with age (Table 2).

For the entire sample, mean values were: dmf-t = 3.05 ± 3.79 (range: 0–20), dmf-s = 6.42 ± 9.58 (range: 0–75).

Girls had slightly higher mean values than boys, but when the t-Welch test was applied, the differences were not statistically significant (Table 3).

Children from rural areas had significantly higher caries experience indices than those from urban areas (Table 4).

Children with special needs also had higher mean values than healthy children, but differences were not statistically significant (Table 5).

### 3.4. Dental Trauma

Of the 602 children, 89 (14.78%) were referred for traumatic dental injuries (48 boys, 41 girls). Children had between 1 and 4 injured teeth, with a mean of 1.66 affected teeth per child.

A total of 148 teeth exhibited trauma: 73 (49.32%) fractures, 75 (50.67%) luxation injuries (Table 6).

### 3.5. Treatments Performed

More than half of the children (55.31%) received a routine check-up with oral hygiene and dietary counseling. Approximately 20% required pulp treatment due to complicated caries.

Restorative treatments (temporary or permanent fillings) were performed in 15.1% of cases. In 6.31% of cases, children were referred for extraction or dental treatment under general anesthesia. Endodontic therapy was performed in 5.14% of cases (Table 7).

## 4. Discussion

Health professionals who interact with young children play an essential role in oral health promotion by providing accurate information and effective support to caregivers. Establishing a dental home as early as possible allows children to benefit from preventive care, such as fluoride application, and guidance regarding home oral hygiene, diet, and trauma prevention. The goal is to educate caregivers and reduce the need for restorative and emergency care later in life [19].

In a multicenter study of more than 2000 children, Nowak et al. (2018) demonstrated that the risk of having caries at the first dental visit increases by a factor of 2.1 for each additional year of age [7]. A child who visits the dentist at age 5 has a 20-fold higher risk of caries compared with a child who attends at age 1 [7]. Similarly, in a study of 2.41 million children under 6 years from 13 US states, Ahmed et al. (2021) reported that the adjusted hazard ratio for caries in children examined for the first time at age 4 was 5.425 times higher than for those examined at age 1 [8].

Early dental visits also reduce lifetime treatment costs [9]. Children who attend dental care during the first year of life have fewer emergency dental needs [6,9], which contributes to lower public health expenditures [20]. Savage et al. followed 9204 children aged ≤5 years for five years and found that those who had their first dental visit by age 1 were more likely to receive preventive services and had nearly 40% lower dental-related costs than children whose first visit occurred at ages 4–5 [5]. Early positive interactions with the dental team also reduce the likelihood of developing dental anxiety [9].

Despite these benefits, many pediatricians refer children to dental care only after age 3. In the United States, 63% of pediatricians refer children after their third birthday, and although 93% of Australian pediatricians report encountering oral health problems, only 40% perform an oral examination [9]. The low rate of early dental visits is strongly associated with poor oral health literacy among caregivers [9].

Consistent with findings from other countries, most children in our study (almost 75%) arrived for their first dental visit at ages 2 or 3, well beyond the recommended age. This delay allowed early lesions to progress into complicated caries, requiring invasive treatment. When the first dental visit occurs at age 2–3 instead of before 12 months, early lesions have months or years to progress from non-cavitated to deep cavitated caries, often resulting in pulp involvement, abscess formation, and the need for pulp therapy, drainage, or treatment under general anesthesia.

Studies from various countries show similar trends: fewer than 3% of children visit the dentist before age 1 [4,12,21,22,23]. In Australia, only 26.7% of children aged 1–2 have ever had a dental check-up [9]. Hartwig et al. (2018) reported that 93.4% of Brazilian children aged 12–18 months had never visited a dentist [11]. Our findings align with these results, showing that two-thirds of infants and toddlers were brought for their first dental visit at ages 2–3.

Studies from the USA, Bulgaria, Poland, Brazil, and Saudi Arabia report mean ages at first dental visit ranging from 3 to 6 years [12,15,21,22,24,25]. In India and Nepal, most children have their first dental visit even later, often after age 5 [26,27,28,29].

Awareness of early dental care is particularly low in rural areas. Rural children tend to seek dental care only when problems become severe. Caregivers often underestimate the importance of primary teeth, fail to recognize early signs of caries, believe children are “too young” for dental visits, or rely on home remedies until pain occurs. These factors contribute to delayed attendance and progression of simple lesions into complicated ones. Atulkar et al. (2015) found that 85% of rural Indian children aged 5–17 had never visited a dentist [26].

Common reasons for delaying the first dental visit include the belief that the child is too young, fear that the child will be scared, underestimation of the importance of primary teeth, and concerns about cost [9,30]. Caregivers often seek dental care for curative rather than preventive reasons [31]. Among children with special needs, only 13% are brought for their first dental visit before age 1 [19]. Barriers include treatment cost, the child’s medical condition, and the willingness of dental providers to treat these patients [19].

In our study, the most common reason for the first dental visit was a routine check-up, although this accounted for less than one-third of cases. Other frequent reasons included non-cavitated and asymptomatic cavitated caries (21.92%) and abscess or swelling (18.43%). Trauma was present in approximately 15% of children. Only a small number were referred for extrinsic discoloration (8%), soft tissue conditions (2%), or neonatal teeth (<1%).

Comparisons with other studies show variability. In Australia, Trinh et al. (2022) reported that 62.7% of children under 3 attended for routine check-ups, a much higher proportion than in our sample [9]. Other reasons included concerns about tooth development (11.8%), injury (7.6%), discoloration (5.9%), and pain (3.4%) [9]. In Saudi Arabia, Alkhuwaiter et al. (2024) found that check-ups and fluoride applications were the most common reasons for first dental visits in children under 3 [32]. In Nigeria, Olatosi et al. (2019) reported different patterns for children under 1 versus those aged 1–3, with trauma being the most common reason in the latter group [23]. In South India, only 3.8% of children had their first dental visit by age 1, mostly due to clefts or natal/neonatal teeth; among children aged 1–3, caries and pain were the main reasons, and trauma was more common in boys [33].

The prevalence of early childhood caries in our study was 57.14%, increasing from 10% in children under 1 year to 78.70% in 3-year-olds. Mean caries experience indices (dmf-t = 3.05 ± 3.79; dmf-s = 6.42 ± 9.58) were higher than values reported in similar studies. For example, Acuna et al. reported ECC prevalence of 4.52% at age 1, 35.54% at age 2, and 34.94% at age 3 in Ecuador [34]. Peric et al. (2025) found a caries prevalence of 44% among Serbian 3-year-olds, with mean dmft = 2.11 ± 3.45 and dfs = 2.62 ± 5.56 [35]. Paiva et al. reported a dmft index of 0.54 in Chilean children under 2 years [36].

Notably, in our study, only 6.68% of children with caries had non-cavitated lesions, while about half already had complicated caries.

Regarding the treatment, 55.31% of children received routine check-ups with oral hygiene and dietary counseling. A total of 19.91% required pulp treatment due to complicated caries. Restorative treatments were performed in 15.1% of cases. In 6.31%, children were referred for treatment under general anesthesia. These findings differ from those of Mika et al. (2018), who reported that 67.5% of Polish children required conservative treatment, 7.8% needed conservative treatment plus extraction, and only 0.6% required extraction alone [22].

In the United States, Medicaid programs reimburse medical providers for oral health assessments and referrals in children under 5. Children who receive preventive oral health care in non-dental settings are less likely to require treatment under general anesthesia. Early dental visits are associated with continued preventive care, fewer restorative needs, and lower costs.

In case of children under 3 years old, the pediatric dentist needs to collaborate with pediatricians and general practitioners to encourage their parents to come for the first dental check-up within the first 6 months after the eruption of the first tooth, as well as for subsequent regular check-ups. Pediatricians and family physicians are the first health professionals who see infants—so if they do not emphasize early dental visits, parentremain unaware of their importance. This is strongly supported by international data: 63% of US pediatricians referred children to a dental professional only after their third birthday and only 40% of Australian pediatricians would perform an oral examination [37,38].

Pediatric dentists, pediatricians, and general practitioners need to work together to raise the parents’ awareness about the oral health status of all infants in order to avoid delayed first dental visits and the occurrence of complicated caries in young children.

### Study Limitations

One of the limitations of the study is that the data were collected from a university clinic, so the place attracts more severe, symptomatic, or emergency cases than a standard local dental practice. Another limitation is that the entire sample is drawn from a single dental care center in Bucharest. The findings cannot accurately represent the diverse geographic regions or the overall pediatric population of Romania. Also, the sample size is heavily skewed toward urban residents (77.41% urban vs. 22.59% rural). This imbalance limits the accuracy and statistical power when comparing the two groups. The study did not take into consideration crucial variables such as parental education, family income, and health insurance status. These factors heavily influence early dental attendance and oral health literacy. There is no data regarding daily oral hygiene habits, home fluoride exposure, or dietary and feeding patterns (such as prolonged nocturnal breastfeeding or bottle-feeding). Relying on existing dental records restricts data collection only to what was previously documented, preventing researchers from asking follow-up questions to clarify parental motivations. Finally, as it is a cross-sectional study, it captures data at a single point in time. It cannot track the long-term clinical outcomes, the success of the treatments applied, or the subsequent compliance of the parents.

## 5. Conclusions

Many infants and toddlers in this study were referred for their first dental visit later than the recommended age, resulting in a high prevalence of caries and a substantial need for therapeutic rather than preventive dental care. More than half of the children presented with caries lesions, and a large proportion already had complicated caries, requiring pulp therapy, abscess management, or referral for treatment under general anesthesia. Significant disparities were observed between rural and urban children from our study sample, with rural children exhibiting higher caries experience indices and more severe disease. Targeted, interdisciplinary public health initiatives are necessary to improve early access to dental care.

## Figures and Tables

**Table 1:** Distribution of patients according to the reason for referral.

The Reason for the First Dental Visit	n	%
Check-up	170	28.23
Presence of asymptomatic caries lesions	132	21.92
Abscess/swelling	111	18.43
Trauma of primary teeth	89	14.78
Extrinsic discoloration	48	7.97
Pain due to caries	36	5.98
Soft tissues conditions	14	2.32
Neonatal teeth	2	0.33

**Table 2:** Caries experience indices by age.

Age Group	n	dmft	dmfs
1–11 months	10	0	0
12–23 months	158	1.40 ± 2.25	2.65 ± 4.92
24–35 months	218	2.61 ± 3.38	5.57 ± 7.90
36–47 months	216	4.83 ± 4.37	10.32 ± 12.15

**Table 3:** Sex distribution of mean values of caries experience indices.

Caries Experience Indices	Boys	Girls	*p*
dmft	2.91 ± 3.66	3.22 ± 3.96	0.33
dmfs	6.24 ± 8.97	6.64 ± 10.34	0.66

**Table 4:** Distribution of mean values of caries experience indices according to residence (* means statistically different).

Caries Experience Indices	Urban Area	Rural Area	*p*
dmft	2.67 ± 3.61	4.33 ± 4.13	0.000 *
dmfs	5.62 ± 8.86	9.16 ± 11.34	0.000 *

**Table 5:** Distribution of mean values of caries experience indices according to the presence or absence of disability.

Caries Experience Indices	Children Without Special Needs	Children with Special Needs	*p*
dmft	3.00 ± 3.75	4.00 ± 4.57	0.166
dmfs	6.36 ± 9.60	7.48 ± 9.41	0.540

**Table 6:** Dental trauma distribution according to their type.

Type of Trauma	n	%
Uncomplicated crown fracture	41	27.70
Complicated crown fracture	16	10.81
Crown-root fracture	12	8.10
Root fracture	4	2.70
Subluxation	10	6.75
Lateral luxation	31	20.94
Extrusive luxation	5	3.37
Intrusive luxation	23	15.54
Avulsion	6	4.05
Total	148	100

**Table 7:** Treatment methods performed during the first dental visit.

	<12 Months	12–23 Months	24–35 Months	36–47 Months	Total
Check-up	8	121	124	80	333 (55.31%)
GIC restoration	0	5	16	21	42 (6.97%)
Interim dental filling	0	5	12	32	49 (8.13%)
Desensitizing pulp therapy	0	0	1	10	11 (1.82%)
Abscess drainage	0	9	31	38	78 (12.95%)
Antibacterial mouthwash	1	6	4	0	11 (1.82%)
Professional dental cleaning	0	1	5	3	9 (1.49%)
Referral for extraction/dental treatment under general anesthesia	1	3	13	21	38 (6.31%)
Endodontic therapy	0	8	12	11	31 (5.14%)
Total	10	158	218	216	602 (100%)

## Data Availability

The data presented in this study are available from the corresponding authors upon reasonable request. The data are not publicly available due to ethical and privacy restrictions involving patient confidentiality and institutional regulations regarding the use of clinical data.
